# Quinoxaline–3-amino­phenol–water (2/1/2)

**DOI:** 10.1107/S1600536808010568

**Published:** 2008-04-23

**Authors:** Agnieszka Czapik, Maria Gdaniec

**Affiliations:** a Faculty of Chemistry, Adam Mickiewicz University, 60-780 Poznań, Poland

## Abstract

The asymmetric unit of the title compound, 2C_8_H_6_N_2_·C_6_H_7_NO·2H_2_O, contains two quinoxaline mol­ecules, one mol­ecule of 3-amino­phenol and two water mol­ecules which are hydrogen bonded to form a two-dimensional polymeric structure. Each of the symmetry-independent quinoxaline mol­ecules forms separate stacks of different symmetry. In one set of stacks, the mol­ecules are related by a screw axis and are slightly tilted [dihedral angle = 7.12 (1)°]. In the second set of stacks, adjacent mol­ecules are parallel and related by an inversion center [inter­planar distances = 3.376 (4) and 3.473 (4) Å].

## Related literature

For supra­molecular ladders, see: Sokolov & MacGillivray (2006 ▶); Sokolov *et al.* (2006 ▶). For complexes of aromatic diaza­heterocycles with phenols, see: Thalladi *et al.* (2000 ▶); Kadzewski & Gdaniec (2006 ▶).
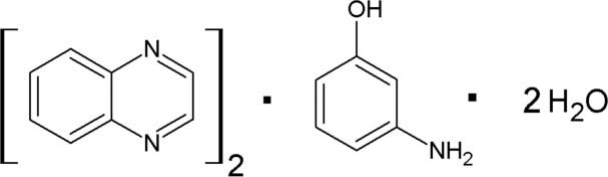


## Experimental

### 

#### Crystal data

2C_8_H_6_N_2_·C_6_H_7_NO·2H_2_O
*M* *_r_* = 405.45
Monoclinic, 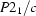

*a* = 15.2951 (10) Å
*b* = 7.1383 (4) Å
*c* = 20.1614 (14) Å
β = 110.775 (8)°
*V* = 2058.1 (3) Å^3^
*Z* = 4
Mo *K*α radiation
μ = 0.09 mm^−1^
*T* = 130.0 (2) K
0.40 × 0.40 × 0.07 mm

#### Data collection

Kuma KM-4-CCD κ-geometry diffractometer
Absorption correction: multi-scan (*CrysAlis RED*; Oxford Diffraction, 2007 ▶) *T*_min_ = 0.966, *T*_max_ = 1.000 (expected range = 0.960–0.994)
16706 measured reflections
3620 independent reflections
2285 reflections with *I* > 2σ(*I*)
*R*_int_ = 0.037

#### Refinement

*R*[*F* ^2^ > 2σ(*F* ^2^)] = 0.031
*wR*(*F* ^2^) = 0.070
*S* = 0.91
3620 reflections
300 parameters
H atoms treated by a mixture of independent and constrained refinement
Δρ_max_ = 0.20 e Å^−3^
Δρ_min_ = −0.14 e Å^−3^

### 

Data collection: *CrysAlis CCD* (Oxford Diffraction, 2007 ▶); cell refinement: *CrysAlis CCD*; data reduction: *CrysAlis RED* (Oxford Diffraction, 2007 ▶); program(s) used to solve structure: *SHELXS97* (Sheldrick, 2008 ▶); program(s) used to refine structure: *SHELXL97* (Sheldrick, 2008 ▶); molecular graphics: *ORTEP-3 for Windows* (Farrugia, 1997 ▶) and *Mercury* (Macrae *et al.*, 2006 ▶); software used to prepare material for publication: *SHELXL97*.

## Supplementary Material

Crystal structure: contains datablocks global, I. DOI: 10.1107/S1600536808010568/fl2194sup1.cif

Structure factors: contains datablocks I. DOI: 10.1107/S1600536808010568/fl2194Isup2.hkl

Additional supplementary materials:  crystallographic information; 3D view; checkCIF report

## Figures and Tables

**Table 1:** Hydrogen-bond geometry (Å, °)

*D*—H⋯*A*	*D*—H	H⋯*A*	*D*⋯*A*	*D*—H⋯*A*
O1*C*—H1*C*⋯N1*B*	0.927 (17)	1.857 (17)	2.7844 (14)	178.7 (16)
N1*C*—H2N*C*⋯O1*E*^i^	0.927 (16)	2.125 (17)	3.0400 (19)	168.8 (14)
N1*C*—H1N*C*⋯O1*D*^ii^	0.891 (16)	2.191 (17)	3.058 (2)	164.4 (13)
O1*D*—H1*D*⋯N1*A*	0.87 (2)	2.01 (2)	2.8651 (17)	166.8 (17)
O1*D*—H2*D*⋯O1*E*^i^	0.94 (2)	1.77 (2)	2.7022 (16)	174.5 (19)
O1*E*—H1*E*⋯O1*D*^iii^	0.95 (2)	1.82 (2)	2.7711 (17)	177.8 (19)
O1*E*—H2*E*⋯N4*A*	0.92 (2)	1.92 (2)	2.8446 (16)	175.4 (18)

Symmetry codes: (i) 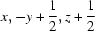; (ii) 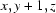; (iii) 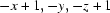.

**Fig. 1.**
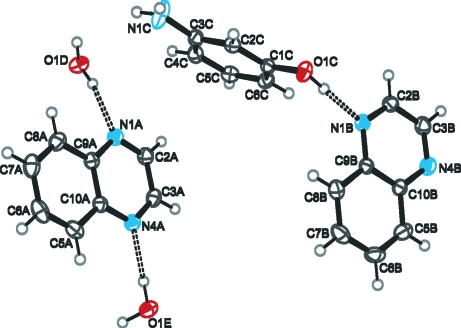
: The molecular structure of the title compound with displacement ellipsoids shown at the 50% probability level. Hydrogen bonds are shown as dashed lines.

**Fig. 2.**
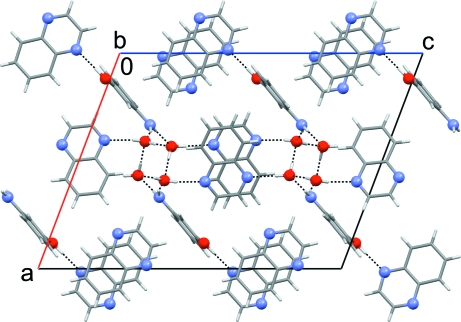
: Crystal packing viewed down the y axis. Hydrogen bonds are shown with dashed lines.

**Fig. 3.**
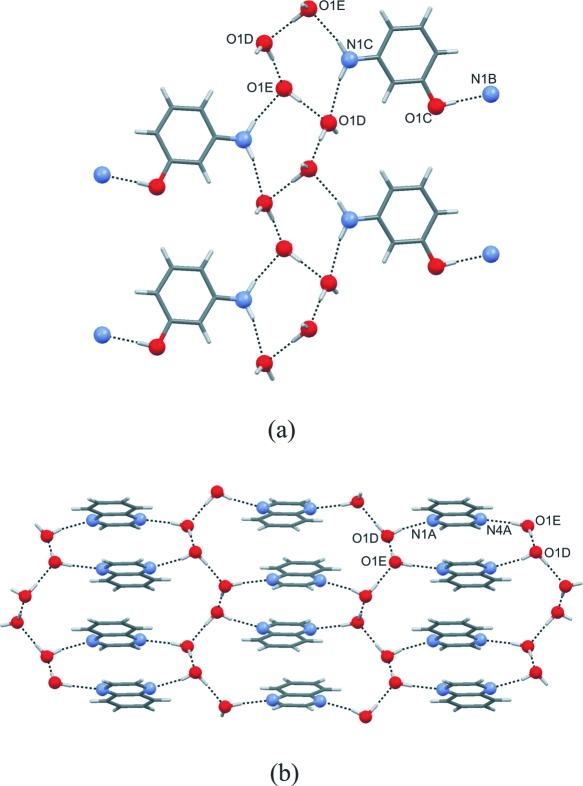
a) the H2O helix with the 3-aminophenol molecules attached to the helix via hydrogen bonds to the amino group, b) two-dimensional polymeric structure formed by hydrogen-bonded quinoxaline A molecules and water molecules.

## supplementary crystallographic information

### Comment

3-Aminophenol shows the ability to direct the assembly of supramolecular ladders *via* hydrogen bonding and π–π stacking interactions in the solid state (Sokolov *et al.*, 2006; Sokolov & MacGillivray, 2006). On the other hand, heterocycles like phenazine and quinoxaline are known to form a robust host framework with one-dimensional channels filled with small aromatic guest molecules (Thalladi *et al.*, 2000; Kadzewski & Gdaniec, 2006). In the course of our studies on molecular complexes of diazaaromatic heterocycles we cocrystallized quinoxaline with 3-aminophenol expecting to obtain ladder-type assemblies analogous to those observed in cocrystals of bipyridines with 3-aminophenol (Sokolov *et al.*, 2006). Unfortunately, the molecular complex with the expected 2:1 component ratio crystallized as a dihydrate (Fig. 1) that had a significant impact on the organization of molecules in the crystal.

Crystal packing of the title compound is shown in Fig. 2. The asymmetric unit contains two quinoxaline molecules, one 3-aminophenol molecule and two water molecules. The water molecules are hydrogen-bonded (for the hydrogen-bond geometry see Table 2) to form a helix extending along the *b* axis with the amino group of the 3-aminophenol linked to the helix *via* N—H···O interactions in the  manner shown in Fig. 3a. The quinoxaline B molecules join to this assembly *via* hydrogen bonds to the phenolic OH groups whereas the quinoxaline A molecules bridge the water helices *via* O—H···N bonding  and π–π stacking interactions generating a supramolecular two dimensional polymeric structure (Figure 3 b). The quinoxaline B molecules are also organized into π–π stacks extending along the *b* axis. The B molecules in the stacks are related by a screw-axis and are slightly tilted [dihedral angle of 7.12 (1)°] whereas the A molecules are parallel and related by inversion centers [interplanar distances of 3.376 (4) and 3.473 (4) Å].

### Experimental

The title compound was obtained by dissolving quinoxaline (0.2 g, 1.54 mmol) and 3-aminophenol (0.084 g, 0.77 mmol) in 5 ml of methanol followed slow evaporation  to yield colorless plates suitable for data collection.

### Refinement

All H atoms were located in electron-density difference maps. C-bonded H atoms were placed at calculated positions, with C—H = 0.93 Å, and were refined as riding on their carrier C atoms, with *U*ĩso~(H) = 1.2Ueq(C). The H atoms of the OH and NH groups were freely refined (coordinates and isotropic displacement parameters).

### Crystal data

**Table:**

2C_8_H_6_N_2_·C_6_H_7_NO·2H_2_O	*F*_000_ = 856
*M**_r_* = 405.45	*D*_x_ = 1.309 Mg m^−^^3^
Monoclinic, *P*2_1_/*c*	Mo *K*α radiation λ = 0.71073 Å
Hall symbol: -P 2ybc	Cell parameters from 5665 reflections
*a* = 15.2951 (10) Å	θ = 2.1–27.9º
*b* = 7.1383 (4) Å	µ = 0.09 mm^−^^1^
*c* = 20.1614 (14) Å	*T* = 130.0 (2) K
β = 110.775 (8)º	Plate, colourless
*V* = 2058.1 (3) Å^3^	0.40 × 0.40 × 0.07 mm
*Z* = 4	

### Data collection

**Table:**

Kuma KM-4-CCD κ-geometry diffractometer	3620 independent reflections
Radiation source: fine-focus sealed tube	2285 reflections with *I* > 2σ(*I*)
Monochromator: graphite	*R*_int_ = 0.037
*T* = 130(2) K	θ_max_ = 25.0º
ω scans	θ_min_ = 4.1º
Absorption correction: multi-scan(CrysAlis RED; Oxford Diffraction, 2007)	*h* = −18→17
*T*_min_ = 0.966, *T*_max_ = 1.000	*k* = −8→8
16706 measured reflections	*l* = −23→23

### Refinement

**Table:**

Refinement on *F*^2^	Hydrogen site location: inferred from neighbouring sites
Least-squares matrix: full	H atoms treated by a mixture of independent and constrained refinement
*R*[*F*^2^ > 2σ(*F*^2^)] = 0.031	*w* = 1/[σ^2^(*F*_o_^2^) + (0.0362*P*)^2^] where *P* = (*F*_o_^2^ + 2*F*_c_^2^)/3
*wR*(*F*^2^) = 0.070	(Δ/σ)_max_ = 0.001
*S* = 0.91	Δρ_max_ = 0.20 e Å^−^^3^
3620 reflections	Δρ_min_ = −0.14 e Å^−^^3^
300 parameters	Extinction correction: SHELXL97 (Sheldrick, 2008), Fc^*^=kFc[1+0.001xFc^2^λ^3^/sin(2θ)]^-1/4^
Primary atom site location: structure-invariant direct methods	Extinction coefficient: 0.0029 (5)
Secondary atom site location: difference Fourier map	

### Special details

**Table:**

Experimental. Absorption correction: SCALE3 ABSPACK scaling algorithm of the Crysalis RED program (Oxford Diffraction, 2007)
Geometry. All e.s.d.'s (except the e.s.d. in the dihedral angle between two l.s. planes) are estimated using the full covariance matrix. The cell e.s.d.'s are taken into account individually in the estimation of e.s.d.'s in distances, angles and torsion angles; correlations between e.s.d.'s in cell parameters are only used when they are defined by crystal symmetry. An approximate (isotropic) treatment of cell e.s.d.'s is used for estimating e.s.d.'s involving l.s. planes.
Refinement. Refinement of *F*^2^ against ALL reflections. The weighted *R*-factor *wR* and goodness of fit *S* are based on *F*^2^, conventional *R*-factors *R* are based on *F*, with *F* set to zero for negative *F*^2^. The threshold expression of *F*^2^ > σ(*F*^2^) is used only for calculating *R*-factors(gt) *etc*. and is not relevant to the choice of reflections for refinement. *R*-factors based on *F*^2^ are statistically about twice as large as those based on *F*, and *R*- factors based on ALL data will be even larger.

### Fractional atomic coordinates and isotropic or equivalent isotropic displacement parameters (Å^2^)

**Table:**

	*x*	*y*	*z*	*U*_iso_*/*U*_eq_	
N1A	0.40385 (8)	0.19751 (16)	0.55810 (6)	0.0257 (3)	
C2A	0.33478 (10)	0.1595 (2)	0.49872 (7)	0.0268 (4)	
H2A	0.2767	0.1284	0.5008	0.032*	
C3A	0.34523 (10)	0.1639 (2)	0.43241 (8)	0.0272 (4)	
H3A	0.2937	0.1354	0.3923	0.033*	
N4A	0.42444 (8)	0.20642 (17)	0.42434 (6)	0.0257 (3)	
C5A	0.58438 (10)	0.3015 (2)	0.48061 (8)	0.0295 (4)	
H5A	0.5917	0.3043	0.4368	0.035*	
C6A	0.65726 (10)	0.3465 (2)	0.54049 (9)	0.0346 (4)	
H6A	0.7144	0.3799	0.5373	0.042*	
C7A	0.64723 (10)	0.3431 (2)	0.60726 (8)	0.0346 (4)	
H7A	0.6977	0.3744	0.6477	0.042*	
C8A	0.56397 (10)	0.2943 (2)	0.61310 (8)	0.0295 (4)	
H8A	0.5578	0.2925	0.6574	0.035*	
C9A	0.48741 (9)	0.24661 (19)	0.55196 (7)	0.0223 (3)	
C10A	0.49775 (9)	0.25055 (19)	0.48513 (7)	0.0215 (3)	
N1B	−0.01606 (8)	0.88628 (16)	0.35287 (6)	0.0237 (3)	
C2B	−0.10425 (10)	0.88544 (19)	0.34628 (7)	0.0257 (4)	
H2B	−0.1194	0.8923	0.3870	0.031*	
C3B	−0.17739 (10)	0.8745 (2)	0.27975 (8)	0.0294 (4)	
H3B	−0.2388	0.8749	0.2785	0.035*	
N4B	−0.16252 (8)	0.86390 (17)	0.21969 (6)	0.0297 (3)	
C5B	−0.04863 (11)	0.8530 (2)	0.16312 (7)	0.0312 (4)	
H5B	−0.0964	0.8453	0.1191	0.037*	
C6B	0.04198 (11)	0.8538 (2)	0.16717 (8)	0.0333 (4)	
H6B	0.0558	0.8487	0.1259	0.040*	
C7B	0.11481 (11)	0.8625 (2)	0.23343 (8)	0.0331 (4)	
H7B	0.1766	0.8617	0.2357	0.040*	
C8B	0.09583 (10)	0.8720 (2)	0.29450 (8)	0.0294 (4)	
H8B	0.1445	0.8775	0.3381	0.035*	
C9B	0.00272 (9)	0.87338 (19)	0.29139 (7)	0.0210 (3)	
C10B	−0.07063 (10)	0.86356 (19)	0.22497 (7)	0.0229 (3)	
C1C	0.15035 (9)	0.7993 (2)	0.52107 (7)	0.0208 (3)	
O1C	0.11852 (7)	0.96292 (14)	0.48513 (5)	0.0286 (3)	
H1C	0.0737 (11)	0.936 (2)	0.4413 (9)	0.061 (6)*	
C2C	0.22485 (9)	0.8116 (2)	0.58454 (7)	0.0217 (3)	
H2C	0.2511	0.9280	0.6007	0.026*	
C3C	0.26114 (9)	0.6520 (2)	0.62471 (7)	0.0240 (4)	
N1C	0.33268 (10)	0.6689 (2)	0.68974 (8)	0.0443 (4)	
H2NC	0.3584 (10)	0.561 (2)	0.7146 (8)	0.044 (5)*	
H1NC	0.3613 (10)	0.780 (2)	0.6997 (8)	0.037 (5)*	
C4C	0.22258 (9)	0.4776 (2)	0.59862 (7)	0.0274 (4)	
H4C	0.2472	0.3690	0.6238	0.033*	
C5C	0.14761 (10)	0.4673 (2)	0.53511 (7)	0.0268 (4)	
H5C	0.1218	0.3509	0.5183	0.032*	
C6C	0.11020 (9)	0.6262 (2)	0.49608 (7)	0.0249 (4)	
H6C	0.0592	0.6176	0.4539	0.030*	
O1D	0.40947 (7)	0.06594 (16)	0.69381 (7)	0.0325 (3)	
H1D	0.4028 (12)	0.121 (3)	0.6539 (11)	0.071 (7)*	
H2D	0.4209 (13)	0.161 (3)	0.7281 (11)	0.089 (8)*	
O1E	0.43284 (8)	0.14985 (16)	0.28706 (6)	0.0320 (3)	
H1E	0.4870 (14)	0.078 (3)	0.2927 (10)	0.086 (7)*	
H2E	0.4315 (12)	0.175 (3)	0.3316 (11)	0.077 (7)*	

### Atomic displacement parameters (Å^2^)

**Table:**

	*U*^11^	*U*^22^	*U*^33^	*U*^12^	*U*^13^	*U*^23^
N1A	0.0264 (7)	0.0255 (8)	0.0254 (7)	−0.0008 (5)	0.0097 (6)	0.0018 (5)
C2A	0.0243 (8)	0.0269 (10)	0.0292 (9)	−0.0017 (7)	0.0095 (7)	0.0011 (7)
C3A	0.0255 (9)	0.0251 (10)	0.0263 (9)	−0.0008 (7)	0.0036 (7)	−0.0005 (7)
N4A	0.0280 (7)	0.0239 (7)	0.0249 (7)	0.0011 (5)	0.0090 (6)	0.0006 (5)
C5A	0.0293 (9)	0.0268 (10)	0.0378 (10)	0.0035 (7)	0.0185 (8)	0.0019 (7)
C6A	0.0228 (9)	0.0256 (10)	0.0568 (12)	−0.0007 (7)	0.0158 (8)	−0.0005 (8)
C7A	0.0274 (9)	0.0274 (10)	0.0396 (10)	0.0002 (7)	0.0001 (8)	−0.0030 (8)
C8A	0.0312 (9)	0.0283 (9)	0.0253 (9)	−0.0006 (7)	0.0054 (7)	−0.0015 (7)
C9A	0.0239 (8)	0.0169 (9)	0.0256 (9)	0.0009 (6)	0.0083 (7)	0.0018 (6)
C10A	0.0247 (8)	0.0155 (9)	0.0248 (9)	0.0030 (6)	0.0094 (7)	0.0005 (6)
N1B	0.0234 (7)	0.0231 (8)	0.0242 (7)	0.0012 (5)	0.0078 (6)	0.0024 (5)
C2B	0.0298 (9)	0.0242 (9)	0.0269 (9)	0.0032 (7)	0.0146 (7)	0.0049 (7)
C3B	0.0218 (8)	0.0331 (10)	0.0348 (10)	0.0012 (7)	0.0120 (7)	0.0063 (7)
N4B	0.0254 (7)	0.0345 (9)	0.0283 (7)	0.0002 (6)	0.0083 (6)	0.0050 (6)
C5B	0.0410 (10)	0.0298 (10)	0.0233 (9)	0.0000 (7)	0.0120 (8)	0.0015 (7)
C6B	0.0486 (11)	0.0281 (10)	0.0331 (10)	0.0024 (8)	0.0266 (8)	0.0046 (7)
C7B	0.0314 (9)	0.0270 (10)	0.0492 (11)	0.0029 (7)	0.0246 (8)	0.0043 (8)
C8B	0.0233 (9)	0.0296 (10)	0.0339 (9)	0.0026 (7)	0.0084 (7)	0.0029 (7)
C9B	0.0241 (8)	0.0164 (8)	0.0235 (8)	0.0019 (6)	0.0099 (7)	0.0027 (6)
C10B	0.0261 (8)	0.0183 (9)	0.0247 (8)	0.0013 (6)	0.0093 (7)	0.0038 (6)
C1C	0.0210 (8)	0.0218 (9)	0.0215 (8)	0.0031 (7)	0.0100 (7)	0.0026 (7)
O1C	0.0286 (6)	0.0246 (7)	0.0255 (6)	−0.0010 (5)	0.0009 (5)	0.0019 (5)
C2C	0.0192 (8)	0.0232 (9)	0.0239 (8)	−0.0042 (6)	0.0088 (6)	−0.0024 (6)
C3C	0.0173 (8)	0.0311 (10)	0.0243 (8)	−0.0010 (7)	0.0082 (7)	0.0038 (7)
N1C	0.0365 (9)	0.0366 (10)	0.0408 (9)	−0.0100 (8)	−0.0099 (7)	0.0144 (8)
C4C	0.0256 (9)	0.0270 (10)	0.0314 (9)	0.0025 (7)	0.0124 (7)	0.0085 (7)
C5C	0.0298 (9)	0.0236 (9)	0.0294 (9)	−0.0049 (7)	0.0136 (7)	−0.0030 (7)
C6C	0.0244 (8)	0.0272 (10)	0.0221 (8)	−0.0031 (7)	0.0069 (7)	−0.0014 (7)
O1D	0.0400 (7)	0.0326 (7)	0.0252 (7)	−0.0059 (5)	0.0119 (5)	−0.0024 (6)
O1E	0.0382 (7)	0.0348 (7)	0.0229 (6)	0.0000 (5)	0.0106 (5)	0.0005 (5)

### Geometric parameters (Å, °)

**Table:**

N1A—C2A	1.3136 (16)	C6B—C7B	1.405 (2)
N1A—C9A	1.3725 (17)	C6B—H6B	0.9300
C2A—C3A	1.402 (2)	C7B—C8B	1.363 (2)
C2A—H2A	0.9300	C7B—H7B	0.9300
C3A—N4A	1.3138 (17)	C8B—C9B	1.4031 (18)
C3A—H3A	0.9300	C8B—H8B	0.9300
N4A—C10A	1.3726 (16)	C9B—C10B	1.4111 (18)
C5A—C6A	1.3597 (19)	C1C—O1C	1.3697 (16)
C5A—C10A	1.4078 (18)	C1C—C2C	1.3818 (17)
C5A—H5A	0.9300	C1C—C6C	1.3919 (19)
C6A—C7A	1.408 (2)	O1C—H1C	0.927 (17)
C6A—H6A	0.9300	C2C—C3C	1.3940 (19)
C7A—C8A	1.365 (2)	C2C—H2C	0.9300
C7A—H7A	0.9300	C3C—N1C	1.3832 (18)
C8A—C9A	1.4080 (18)	C3C—C4C	1.398 (2)
C8A—H8A	0.9300	N1C—H2NC	0.927 (16)
C9A—C10A	1.4116 (19)	N1C—H1NC	0.891 (16)
N1B—C2B	1.3077 (16)	C4C—C5C	1.3850 (18)
N1B—C9B	1.3711 (17)	C4C—H4C	0.9300
C2B—C3B	1.4114 (19)	C5C—C6C	1.3839 (19)
C2B—H2B	0.9300	C5C—H5C	0.9300
C3B—N4B	1.3112 (18)	C6C—H6C	0.9300
C3B—H3B	0.9300	O1D—H1D	0.87 (2)
N4B—C10B	1.3714 (16)	O1D—H2D	0.94 (2)
C5B—C6B	1.3591 (19)	O1E—H1E	0.95 (2)
C5B—C10B	1.404 (2)	O1E—H2E	0.92 (2)
C5B—H5B	0.9300		
			
C2A—N1A—C9A	116.37 (12)	C5B—C6B—H6B	119.8
N1A—C2A—C3A	122.51 (14)	C7B—C6B—H6B	119.8
N1A—C2A—H2A	118.7	C8B—C7B—C6B	120.66 (14)
C3A—C2A—H2A	118.7	C8B—C7B—H7B	119.7
N4A—C3A—C2A	123.05 (13)	C6B—C7B—H7B	119.7
N4A—C3A—H3A	118.5	C7B—C8B—C9B	119.86 (14)
C2A—C3A—H3A	118.5	C7B—C8B—H8B	120.1
C3A—N4A—C10A	116.07 (12)	C9B—C8B—H8B	120.1
C6A—C5A—C10A	119.83 (15)	N1B—C9B—C8B	119.66 (12)
C6A—C5A—H5A	120.1	N1B—C9B—C10B	120.68 (12)
C10A—C5A—H5A	120.1	C8B—C9B—C10B	119.65 (13)
C5A—C6A—C7A	120.79 (15)	N4B—C10B—C5B	119.54 (13)
C5A—C6A—H6A	119.6	N4B—C10B—C9B	121.44 (13)
C7A—C6A—H6A	119.6	C5B—C10B—C9B	119.02 (13)
C8A—C7A—C6A	120.54 (14)	O1C—C1C—C2C	117.20 (13)
C8A—C7A—H7A	119.7	O1C—C1C—C6C	122.48 (12)
C6A—C7A—H7A	119.7	C2C—C1C—C6C	120.32 (13)
C7A—C8A—C9A	119.86 (14)	C1C—O1C—H1C	109.2 (11)
C7A—C8A—H8A	120.1	C1C—C2C—C3C	120.90 (13)
C9A—C8A—H8A	120.1	C1C—C2C—H2C	119.6
N1A—C9A—C8A	119.65 (13)	C3C—C2C—H2C	119.6
N1A—C9A—C10A	120.96 (12)	N1C—C3C—C2C	119.84 (14)
C8A—C9A—C10A	119.40 (13)	N1C—C3C—C4C	121.36 (14)
N4A—C10A—C5A	119.40 (13)	C2C—C3C—C4C	118.78 (13)
N4A—C10A—C9A	121.01 (13)	C3C—N1C—H2NC	118.7 (10)
C5A—C10A—C9A	119.59 (13)	C3C—N1C—H1NC	116.8 (10)
C2B—N1B—C9B	116.56 (11)	H2NC—N1C—H1NC	122.4 (14)
N1B—C2B—C3B	122.56 (14)	C5C—C4C—C3C	119.71 (13)
N1B—C2B—H2B	118.7	C5C—C4C—H4C	120.1
C3B—C2B—H2B	118.7	C3C—C4C—H4C	120.1
N4B—C3B—C2B	122.83 (14)	C6C—C5C—C4C	121.45 (14)
N4B—C3B—H3B	118.6	C6C—C5C—H5C	119.3
C2B—C3B—H3B	118.6	C4C—C5C—H5C	119.3
C3B—N4B—C10B	115.92 (12)	C5C—C6C—C1C	118.79 (13)
C6B—C5B—C10B	120.46 (14)	C5C—C6C—H6C	120.6
C6B—C5B—H5B	119.8	C1C—C6C—H6C	120.6
C10B—C5B—H5B	119.8	H1D—O1D—H2D	106.6 (18)
C5B—C6B—C7B	120.34 (14)	H1E—O1E—H2E	107.9 (16)

### Hydrogen-bond geometry (Å, °)

**Table:**

*D*—H···*A*	*D*—H	H···*A*	*D*···*A*	*D*—H···*A*
O1C—H1C···N1B	0.927 (17)	1.857 (17)	2.7844 (14)	178.7 (16)
N1C—H2NC···O1E^i^	0.927 (16)	2.125 (17)	3.0400 (19)	168.8 (14)
N1C—H1NC···O1D^ii^	0.891 (16)	2.191 (17)	3.058 (2)	164.4 (13)
O1D—H1D···N1A	0.87 (2)	2.01 (2)	2.8651 (17)	166.8 (17)
O1D—H2D···O1E^i^	0.94 (2)	1.77 (2)	2.7022 (16)	174.5 (19)
O1E—H1E···O1D^iii^	0.95 (2)	1.82 (2)	2.7711 (17)	177.8 (19)
O1E—H2E···N4A	0.92 (2)	1.92 (2)	2.8446 (16)	175.4 (18)

Symmetry codes: (i) *x*, −*y*+1/2, *z*+1/2; (ii) *x*, *y*+1, *z*; (iii) −*x*+1, −*y*, −*z*+1.
